# Systemic delivery of targeted nanotherapeutic reverses angiotensin II-induced abdominal aortic aneurysms in mice

**DOI:** 10.1038/s41598-021-88017-w

**Published:** 2021-04-21

**Authors:** Xiaoying Wang, Vaideesh Parasaram, Saphala Dhital, Nasim Nosoudi, Shahd Hasanain, Brooks A. Lane, Susan M. Lessner, John F. Eberth, Naren R. Vyavahare

**Affiliations:** 1 Department of Bioengineering, Clemson University, 501 Rhodes Engineering Research Center, Clemson, SC 29634 USA; 2 Biomedical Engineering, College of Engineering & Computer Sciences, Marshall University, Huntington, WV USA; 3 Department of Cell Biology and Anatomy, University of South Carolina School of Medicine, Columbia, USA

**Keywords:** Cardiology, Cardiovascular diseases, Biomaterials, Nanobiotechnology, Regenerative medicine

## Abstract

Abdominal aortic aneurysm (AAA) disease causes dilation of the aorta, leading to aortic rupture and death if not treated early. It is the 14th leading cause of death in the U.S. and 10th leading cause of death in men over age 55, affecting thousands of patients. Despite the prevalence of AAA, no safe and efficient pharmacotherapies exist for patients. The deterioration of the elastic lamina in the aneurysmal wall is a consistent feature of AAAs, making it an ideal target for delivering drugs to the AAA site. In this research, we conjugated nanoparticles with an elastin antibody that only targets degraded elastin while sparing healthy elastin. After induction of aneurysm by 4-week infusion of angiotensin II (Ang II), two biweekly intravenous injections of pentagalloyl glucose (PGG)-loaded nanoparticles conjugated with elastin antibody delivered the drug to the aneurysm site. We show that targeted delivery of PGG could reverse the aortic dilation, ameliorate the inflammation, restore the elastic lamina, and improve the mechanical properties of the aorta at the AAA site. Therefore, simple *iv* therapy of PGG loaded nanoparticles can be an effective treatment option for early to middle stage aneurysms to reverse disease progression and return the aorta to normal homeostasis.

## Introduction

Abdominal aortic aneurysms (AAAs) are characterized by chronic transmural inflammation^1^, resulting in the breakdown of extracellular matrix proteins such as elastin and collagen in the aortic wall, and this plays a vital role in AAA pathophysiology^2^. Mounting evidence has suggested that elastin, one of the key components of the extracellular matrix (ECM), drives progression of this disease by virtue of its degradation by matrix metalloproteinases (MMPs) at the disease site^3^.

AAAs, due to their asymptomatic nature, are only detected during general screening^4^; surgical intervention is only recommended when the diameter of the aorta grows to 5.5 cm or larger, as the risk outweighs benefits below those levels in these patients. No pharmacotherapy is available for those patients who are diagnosed with small AAA. Some small AAAs do rupture and cause death; therefore, pharmacotherapy to prevent AAA's further growth is needed. An ideal therapeutic intervention would prevent further ECM degradation and restore structural integrity and cellular homeostasis, leading to reversing the disease. Our group has successfully demonstrated the beneficial effects of pentagalloyl glucose (PGG) in stabilizing and regenerating vascular ECM both in vitro and in vivo^5^.

While no single animal model captures AAA disease comprehensively, the angiotensin II infusion model shows the spontaneous development of AAAs with very similar pathophysiology to human AAA. Here we present a successful treatment of AAAs in the Ang II model using targeted nanoparticle delivery. We show that PGG-loaded bovine serum albumin (BSA) nanoparticles can be targeted to damaged elastin in aneurysmal mouse aorta. Delivered PGG not only protects elastin from MMP-mediated degradation but also regenerates lost elastic lamellae. PGG delivery also triggered anti-inflammatory signals locally and systemically. Using inflation-extension testing, we further demonstrate the efficacy of the treatment in restoring aortic mechanical properties.

## Materials and methods

### Preparation of bovine serum albumin (BSA) NPs

BSA NPs were prepared using the coacervation method described previously^5^. PGG-loaded NPs (PGG-NPs) were prepared for the treatment study using a modified version of the procedure described above. 250 mg of BSA (Seracare, Milford, MA) was dissolved in 4 mL of DI water. 125 mg PGG (Ajinomoto Omnichem) was dissolved in 400 µl of dimethyl sulfoxide and added slowly to the BSA solution under stirring. Glutaraldehyde (37 µL of 8%, EM grade) was then added as a crosslinker. After an hour of stirring at room temperature, the mixture was added dropwise to 24 mL of ethanol (Sigma, St. Louis, MO) under continuous sonication (Omni Ruptor 400 Ultrasonic Homogenizer, Omni International Inc, Kennesaw, GA) on ice for 30 min. PGG-NPs were obtained by centrifugation at 6000 rpm for 10 min and washed with water by resuspension. Blank (BLN) nanoparticles were prepared by omitting PGG addition.

For targeting visualization, 1, 1-dioctadecyl-3, 3, 3, 3-tetramethylindotricarbocyanine iodide (DiR) dye (PromoCell GmbH, Heidelberg, Germany) loaded BSA NPs (DiR-NPs) were produced for studying in-vivo targeting using the procedure described above with modifications. 250 mg of BSA was dissolved in 4 ml of deionized (DI) water. 2.5 mg of DiR dye was dissolved in acetone first and was then added to the BSA solution. 10.5 mg glutaraldehyde was added as a crosslinker during stirring. After an hour of stirring at room temperature, the mixture was added dropwise to 24 mL of ethanol, under continuous sonication on ice for 30 min. DiR-NPs were obtained by centrifugation at 4000 rpm for 10 min and washed with water three times by resuspension.

### Conjugation of NPs with an anti-elastin antibody

All NPs were conjugated with antibodies before being used in the animal study as described previously^5^. Briefly, 10 mg of NPs (blank, DiR-loaded, or PGG-loaded) were PEGylated with 2.5 mg α-maleimide-ω-N-hydroxysuccinimide ester poly(ethylene glycol) (mPEG-NHS, M.W. 2000, Nanocs, NY, U.S.A.) at room temperature for one hour under gentle vortexing. 68 μg of Traut’s reagent (G-Biosciences, Saint Louis, MO) was used for thiolation of 20 μg of an in house-made elastin antibody (EL)^6^, and the mixture was incubated in 4-(2-hydroxyethyl)-1-piperazineethanesulfonic acid (HEPES) buffer (20 mM, pH = 9.0) for an hour at room temperature under gentle vortexing. Thiolated antibodies were added to the PEGylated NPs and incubated overnight at 4 °C on a slow rocker for conjugation.

### Nanoparticle therapy in Angiotensin II (Ang II) mouse model

Forty-two male low density-lipoprotein receptor-deficient (LDLr −/−) mice (2 months of age, on a C57BL/6 background) were obtained from the Jackson Laboratory (Bar Harbor, ME) for investigating the effects of NP treatment on aneurysms. Aneurysms were induced by systemic infusion of Ang II (Bachem Americas, Torrance, CA) in combination with a diet with saturated fat (21% wt/wt) and cholesterol (0.2% wt/wt; catalog no. TD88137; Harlan Teklad) throughout the whole study. Osmotic pumps (model 2004; Alzet, Cupertino, CA) filled with Ang II were implanted subcutaneously into the mice. Mice were anesthetized by inhalation of 2–3% isoflurane during the surgery. The osmotic pump released Ang II consistently for 4 weeks at a rate of 1000 ng/kg/min. Disease progression was monitored with a Fujifilm VisualSonics Vevo 2100 ultrasound machine (Fujifilm VisualSonics, Toronto, ON, Canada) by utilizing a linear array probe (MS-550D, broadband frequency 22 MHz–55 MHz).

**Figure 1:**
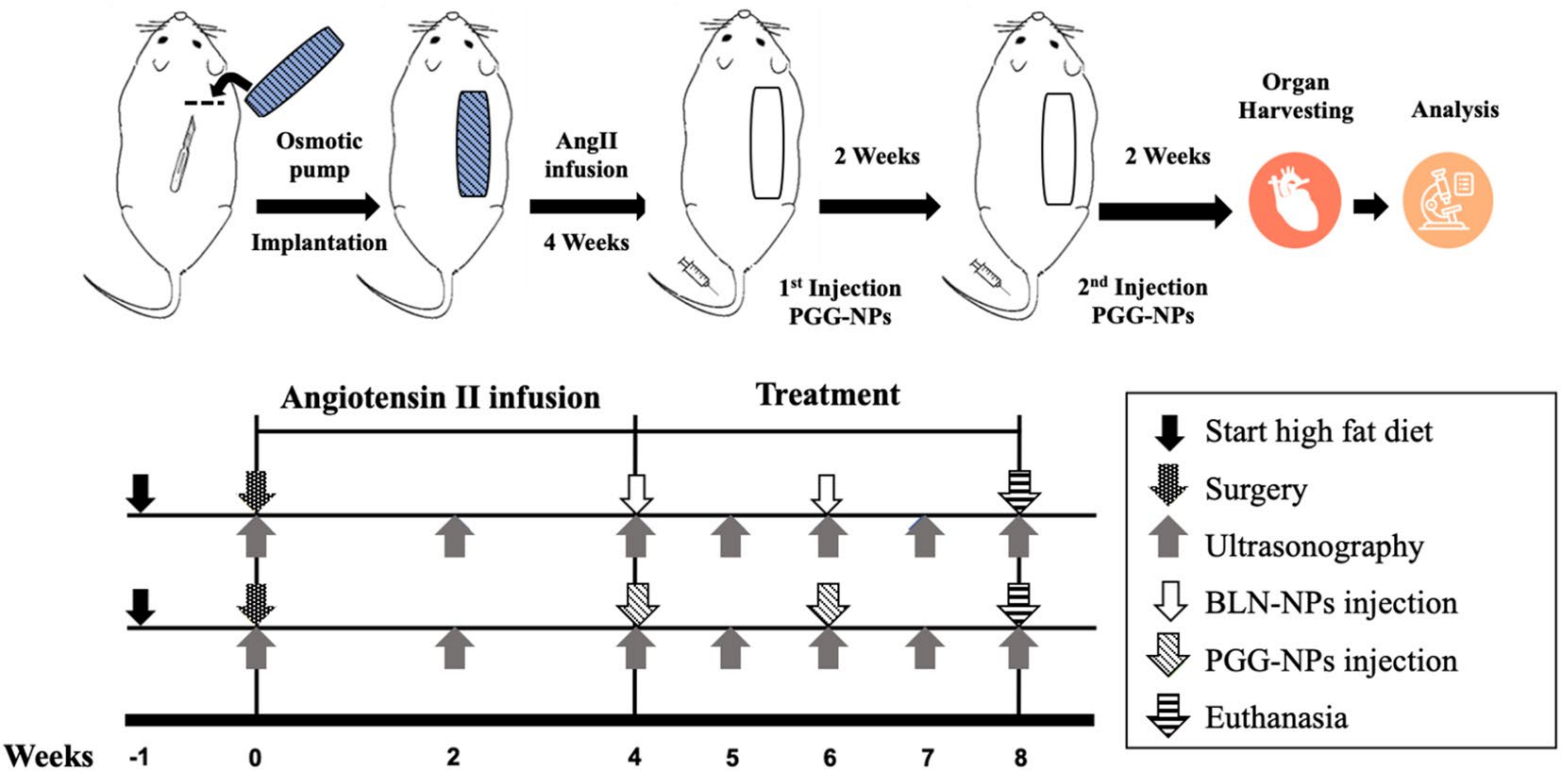
Schematic representation of the study design (created in powerpoint).

For the targeting study, DiR-NPs (10 mg/kg animal weight) were injected intravenously into three mice after the 4-week infusion of Ang II. These mice were euthanized (CO_2_ asphyxiation) 24 h later, and the aortas were harvested for further analysis. For the treatment study, twelve mice were treated with EL- conjugated blank NPs while another twelve mice received EL-conjugated PGG-NPs, both at a dose of 10 mg/kg animal weight, at week 4 and week 6 after Ang II infusion via the tail vein. Fifteen mice that did not receive any treatment were used as controls. Three of the control mice were euthanized at week 4; all other mice were euthanized at week eight (CO_2_ asphyxiation), as shown in the timeline graph (Fig. 1).

For the Ang II model, mice were fed with high fat diet for 1 week before implantation of Ang II-filled osmotic pumps. Elastin antibody conjugated BLN-NPs or PGG-NPs were injected into different mouse groups intravenously at week 4 and week 6 after osmotic pump implantation. At the end of the experiment (8 week), animals were euthanized, and organs were harvested for histological and mechanical tests. Ultrasounds of AAA were carried out every week.

Blood was collected via a heart puncture, and organs were harvested after being perfused with saline for further analysis. In addition, the Ang-II infused LDLr−/− controls CTRL (n = 5) and Ang-II infused LDLr−/− pentagalloyl glucose (PGG)-treated groups (n = 5), 12–14-week-old wild-type C57BL/6 mice (n = 5) were used as healthy (HLTY) controls for ex vivo biomechanical experiments.

All animal use protocols for the study were approved by the Institutional Animal Care and Use Committee (IACUC) at Clemson University (protocol number 2019-018). Clemson University animal facility is AAALC accredited. The animal studies complied with ARRIVE guidelines.

### Ultrasound analysis of the aneurysms

The ultrasound system was used for monitoring percent dilation, circumferential strain, and pulse wave velocity (PWV) throughout the cardiac cycle once before the surgery, biweekly during the Ang II infusion period, and weekly during the treatment study. The animals were placed in a supine position on the imaging table and were maintained under anesthesia by inhaling 2 to 3% isoflurane during imaging. The heart rate and body temperature of the mice were carefully monitored. The heart rate was controlled between 400 ~ 450 BPM and their normal body temperature was maintained using a heating pad. Suprarenal aortic inner diameters were measured using the built-in ultrasound software on the brightness (B)-mode ultrasound images. The percentage of dilation was calculated using the equation given below,

1 $$Dilation (\%)=({\stackrel{-}{D}}_{L}-{\stackrel{-}{D}}_{H})/{\stackrel{-}{D}}_{L}\times 100$$

where ${\stackrel{-}{D}}_{H}$ represents the mean inner diameter of the suprarenal aorta before the surgery and ${\stackrel{-}{D}}_{L}$ is the mean inner diameter after surgery at each timepoint.

Motion (M)-mode ultrasound images of the suprarenal aorta of each mouse were obtained and analyzed to calculate the circumferential strain. Systolic and diastolic diameters were measured and recorded at three different regions in each aneurysm using the built-in ultrasound software. The diastolic-to-systolic circumferential Green–Lagrange strains (CS) were calculated assuming axial symmetry using the equation given below,

2 $$CS=(1/2)({({\stackrel{-}{D}}_{sys}/{\stackrel{-}{D}}_{dia})}^{2}-1)$$

where ${\stackrel{-}{D}}_{sys}$ represents the mean inner diameter of the suprarenal aorta at systole and ${\stackrel{-}{D}}_{dia}$ the mean inner diameter at diastole.

Furthermore, the percentage change of circumferential strain throughout the study was calculated with the following equation,

3 $$CS (\%)=({CS}_{L}-{CS}_{H})/{CS}_{L}\times 100$$

where ${CS}_{H}$ represents the circumferential strain of the suprarenal aorta before the surgery and ${CS}_{L}$ is the circumferential strain after surgery at each timepoint.

Pulse Wave (PW) Doppler mode was used to measure the blood flow at the beginning and the endpoint of the suprarenal aortic lumen prior to each PWV acquisition. All PW Doppler measurements were performed at a Pulse Repetition Frequency (PRF) of 20 kHz and a 45° incidence angle. Throughout the acquisition, the electrocardiogram (ECG) for each mouse was monitored and recorded. PWV was then calculated with the equation below,

4 $$PWV=z/({t}_{2}-{t}_{1})$$

where *z* represents the proximal to distal length of the suprarenal aorta, ${t}_{1}$ is the time when pressure wave arrives at the beginning point and ${t}_{2}$ is the time when pressure wave arrives at the endpoint referenced to the R wave of the ECG.

The percentage change of PWV throughout the study was calculated with the following equation,

5 $$PWV (\%)=({PWV}_{L}-{PWV}_{H})/{PWV}_{L}\times 100$$

where ${PWV}_{H}$ represents the PWV of the suprarenal aorta before the surgery and ${PWV}_{L}$ is the PWV after surgery at the time points mentioned above.

### Histological analysis of the aneurysms

Frozen aortas were embedded in optimal cutting temperature (OCT) compound (Sakura Finetek, Torrance, CA) after being washed in DI water and then sectioned per standard procedures. Five-micrometer sections were mounted on positively-charged glass slides. Slides were placed in 100% pre-cooled acetone (Fisher Science Education, Nazareth, PA) for 10 min to fix the sections. Subsequently, the slides were rinsed with tap water for 5 min to remove the OCT compound and stained with hematoxylin and eosin (H&E) for tissue morphology and with Verhoeff-van Gieson (VVG) for elastic fibers. Some fresh samples were imaged directly after removing the OCT compound and imaged with a fluorescence microscope to visualize the DiR-NPs targeting within the tissue.

### Systemic interferon-gamma (IFN-γ) levels

Blood was drawn via a heart stick with a 1 mL syringe and allowed to clot for 30 min. The serum was collected by centrifuging at 14,000 rpm for ten minutes at 4 °C. IFN-γ amount was quantified from the mouse serum using the standard from the commercial mouse ELISA kit (Invitrogen, Carlsbad, CA).

### Flow cytometry

The spleens and thymuses of the mice from BLN-NPs, PGG-NPs, and control groups were collected, minced, and meshed on 70 μm filters. Immunofluorescence staining was performed as described in a previous study^7,8^. Briefly, cells were incubated with Fc block solution (purified anti-mouse CD16/CD32, clone 2.4G2, BD Biosciences) for 15 min at room temperature to prevent non-specific binding. For cell surface markers, cells were incubated with a fluorescently conjugated antibody against CD 68, APC anti-CD 68 (clone FA-11) (BioLegend, San Diego, CA), in the dark for 30 min at 4 °C. After washing of cells in fluorescence-activated cell sorting (FACS) buffer, flow cytometric data acquisition was performed using an Attune NxT flow cytometer (Thermo Fisher Scientific, Waltham, MA). Data analysis was performed using FlowJo (TreeStar, Ashland, OR) software.

### In-situ zymography

*In-situ* zymography was performed on 5 μm frozen sections to evaluate MMP activity in the suprarenal aortic tissue samples. All cryosections were rinsed with tap water for 5 min to remove the OCT. Each section was treated with 200 μL of a mixture of 0.2 mg/mL DQ-Gelatin (Invitrogen, Eugene, OR) in a developing buffer and then incubated at 37 °C for two hours. After being washed in DI water for 5 min, all sections were stained with DAPI (Life Technologies, Carlsbad, CA) for 5 min and an EVOS XL cell imaging system (Invitrogen, Bothell, WA) was used to capture images of the samples.

### Immunohistochemistry (IHC) for macrophages

All cryosections were rinsed with PBS 3 times for 5 min each time to remove the OCT compound. The sections were first treated with 3% hydrogen peroxide at room temperature for 10 min to block endogenous peroxidase activity. Then, the nonspecific binding sites on the sections were blocked with ‘Background Buster’ (Innovex Biosciences, Richmond, CA) at room temperature for 30 min. The sections were incubated with the primary antibody (anti-CD68 or anti-CD80) at 4 °C overnight. An avidin–biotin complex method (Vector, Burlingame, CA) was used to detect and amplify the target signal, and the staining was completed using a DAB kit (Vector, Burlingame, CA). Slides were counterstained with hematoxylin.

### Quantitative polymerase chain reaction (qPCR)

Suprarenal aortas were homogenized and lysed in TRIzol (Invitrogen, Carlsbad, CA) for mRNA extraction. mRNAs were isolated per the manufacturer’s protocol. The quality of the mRNA extracted was confirmed by measuring the A260/A280 ratio using a BioTek Synergy 2 plate reader (BioTek, Winooski, VT). Primers were designed for amplification of MMP-2, tissue inhibitor of metalloproteinase (TIMP)-1, TIMP-2, and housekeeping genes actin-beta (ACTB) and peptidylprolyl isomerase A (PPIA) (sequences are shown in Table 1) and obtained from Integrated DNA Technologies (Coralville, IA). cDNA was obtained via reverse transcription (RT) using a OneStep RT-PCR kit (Qiagen, Valencia, CA) as per manufacturer’s protocol. qPCR amplification was performed in the following sequence using a Rotor gene qPCR machine (Qiagen, Valencia, CA): 15 s at 94 °C, 30 s at 60 °C, and 30 s at 72 °C for 40 cycles. Gene expression in each sample was normalized to the expression of the housekeeping genes and compared to the control group using the 2^−(ΔΔT)^ cycle threshold (Ct) analysis as follows:

**Table 1:** Primers designed for qPCR.

MMP-2	Mouse MMP2_FWD	AACGGTCGGGAATACAGCAG
	Mouse MMP2_REV	GTAAACAAGGCTTCATGGGGG
TIMP-1	Mouse TIMP1_FWD	GGCATCTGGCATCCTCTTGT
	Mouse TIMP1_REV	TTAGCATCCAGGTCCGAGTTG
TIMP-2	Mouse TIMP2_FWD	TATCTACACGGCCCCCTCTT
	Mouse TIMP2_REV	TCCCAGGGCACAATGAAGTC

6 $$\Delta \Delta \mathrm{Ct}={\left({\mathrm{Ct}}_{\mathrm{Target\, gene}}-{\mathrm{Ct }}_{\mathrm{Reference\, gene}}\right)}_{Experimental}-{({\mathrm{Ct}}_{\mathrm{Target\, gene}}-{\mathrm{Ct }}_{\mathrm{Reference\, gene}})}_{Control}$$

### Biomechanical testing of suprarenal aortas

The mechanical properties of healthy (HLTY), aneurysmal control (CTRL), and PGG-treated (PGG) suprarenal aortas were tested at the University of South Carolina (U of SC) within 48 h of animal euthanasia on a custom-designed murine-specific mechanical testing device^9,10^. Briefly, the device was equipped with a 108 mN thin-beam load cell, 260 mmHg pressure transducer, motorized inflation syringe pump, 1280 × 1024 pixel CMOS camera with 0.5–2 × imaging lens, and a linear actuator with a controller. All components have a USB interface and are synchronously controlled and/or data recorded using a PC running a custom-written LabView code. Vessels were mounted to their fixtures within a polycarbonate testing chamber filled with warm PBS solution and then the unloaded outer diameter and length were recorded using optical calipers.

The arteries underwent five preconditioning cycles consisting of pressurization and stretching from 10 to 140 mmHg at roughly the force-invariant axial stretch ratio. For data acquisition, the artery then underwent equivalent pressurization and stretching cycles with outer diameter measurements taken at 10 mmHg pressure increments. Data was recorded in triplicate, and the average of these tests was used. Primary continuous data collection consisted of pressure, stretch, and deformed outer diameter at the peak bulge location. After mechanical testing, vessels were pressure fixed by replacing luminal PBS with 4% paraformaldehyde solution and re-inflated to 100 mmHg for 4 h. Micro-computed tomography imaging (Quantum GX Micro-CT Imaging System; PerkinElmer) was used to measure the deformed inner diameter on tissues fixed in the physiologically pressurized and axially extended state. Measurements consisted of an average of 4 locations along the circumference of the aneurysm bulge and were processed using Fiji-ImageJ.

To facilitate data acquisition, we assumed axial symmetry and incompressibility of cylindrical segments so that the inner diameter could be calculated from micro-CT-based volume measurements during real-time mechanical testing. From the primary data, the mid-wall circumferential stretch ratio and mean circumferential Cauchy stress are calculated by

7 $$\lambda = \frac{{{d_i} + {d_o}}}{{{D_i} + {D_o}}},\sigma = P\frac{{d_i}}{{{d_o} - {d_i}}},$$

where $P$ is the transmural pressure, ${D_i}$ and ${D_o}$ is the unloaded inner and outer diameter respectively, and ${d_i}$ and ${d_o}$ the corresponding diameters in the loaded configuration. The tangential stiffness, a linearized measure of stiffness, was calculated from the change in stress and the corresponding change in stress (i.e., ${{\Delta \sigma } \mathord{\left/ {\vphantom {{\Delta \sigma } {\Delta \lambda }}} \right. \kern-\nulldelimiterspace} {\Delta \lambda }}$) at a point along the stress-stretch curve. In our study, we chose two distinct configurations to measure the tangential stiffness, namely one that corresponds to 100 mmHg (thus different stretches for each sample), and a common but low stretch of 1.1.

### Statistical analysis

The data are reported as the mean ± standard deviation. Normality and equivalent variance for all data sets were tested before further analysis. Data from the same group at different time points were analyzed by repeated measures ANOVA followed by Tukey’s HSD as a post-hoc test. Data from different groups were analyzed by one-way analysis of variance (ANOVA) followed by Tukey’s HSD as a post-hoc test.

## Results

### Ang II infusion-induced mouse model and in vivo targeting study

After four weeks of Ang II infusion, varying degrees of aneurysms were found in the suprarenal region of the mouse aortas. Based on ultrasound diameter measurements at week four, the mice with dilations of more than 100% at the suprarenal aorta were used for further study.

**Figure 2:**
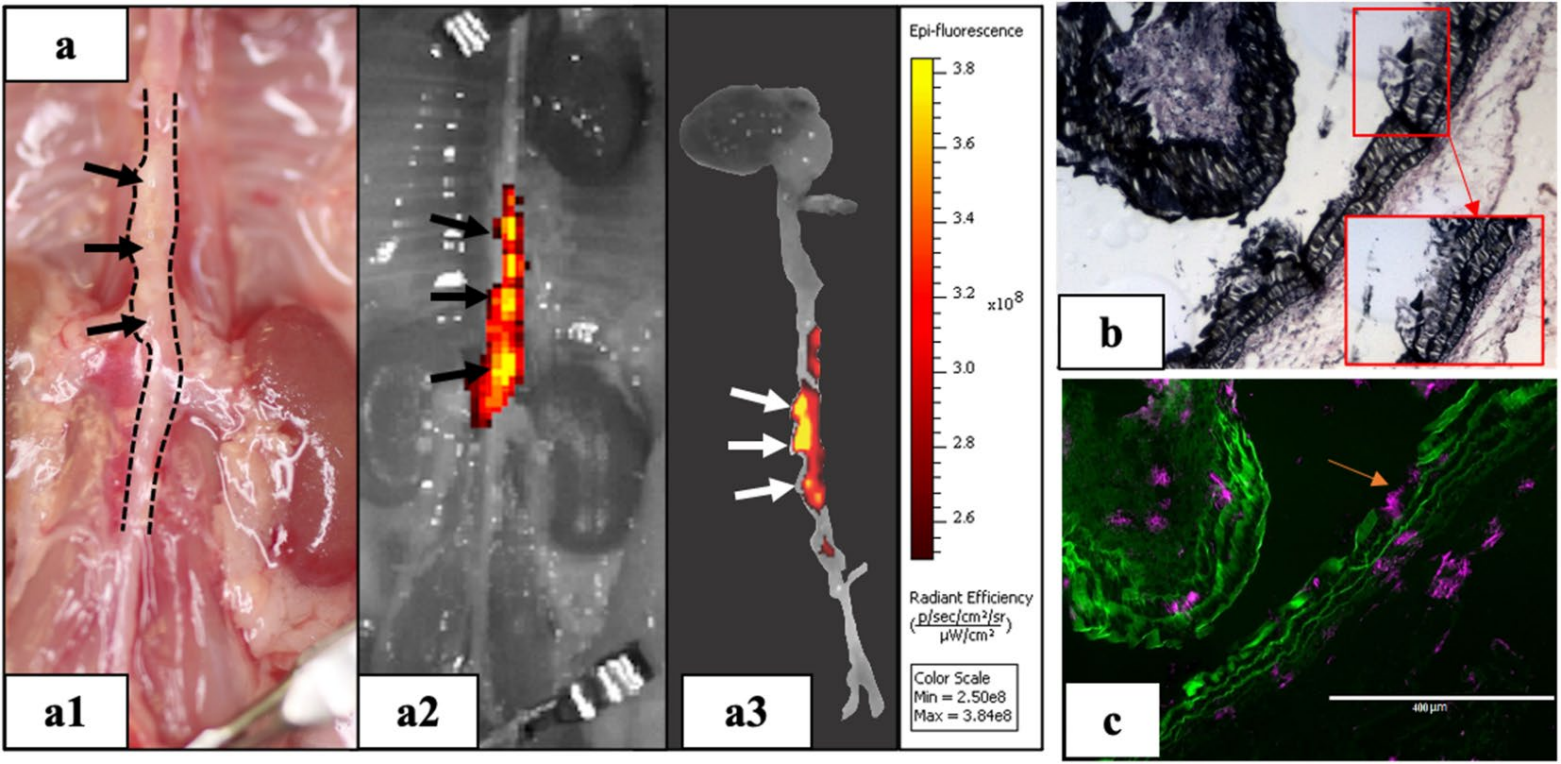
DiR-NPs targeting in Ang II mouse model. (**a**) In-vivo targeting study using DiR loaded nanoparticles (DiR-NPs). DiR NPs were injected via tail vein and after 24 h animals were euthanized d to assess the targeting. The signal given by the DiR-NPs in the IVIS images (a2,a3) indicated that DiR-NPs only accumulate at the suprarenal aortic area where the aneurysm developed (a1), suggesting the successful targeting of the nanoparticles to the aneurysmal tissue; (**b**) VVG staining of the aneurysmal tissue showing elastin damage and (**c**) fluorescent image of the same site showing distribution of the DiR-NPs (purple) within the tissue, indicating that DiR-NPs target the degraded elastin (green autofluorescence) in the aneurysm.

To study the in vivo targeting of the nanoparticle carrier, DiR-NPs were delivered systemically via tail vein and allowed to circulate for 24 h. The aortas of the mice were explanted and imaged ex-vivo using the IVIS Lumina imaging system (PerkinElmer, Branford, CT) to visualize the signal given by the DiR-NPs (Fig. 2). According to the IVIS images (Fig. 2a2,a3), the suprarenal region of the mouse aortas where the aneurysms were found (Fig. 2a1) showed a high fluorescent signal exclusively, indicating accumulation of the DiR-NPs in the aneurysmal tissue. Further histological analysis showed that DiR-NPs appeared at the sites where degraded elastin was displayed in the VVG staining (Fig. 2b,c). We also did histological staining for descending thoracic aorta away from the aneurysm site and found no accumulation of DiR-NPs (supplemental Fig. 1). These results confirmed that our nanoparticle carrier could successfully reach and accumulate in the aneurysmal tissue by targeting degraded elastin in the ECM.

### Influence of PGG nanoparticle treatment on aortic dilation

**Figure 3:**
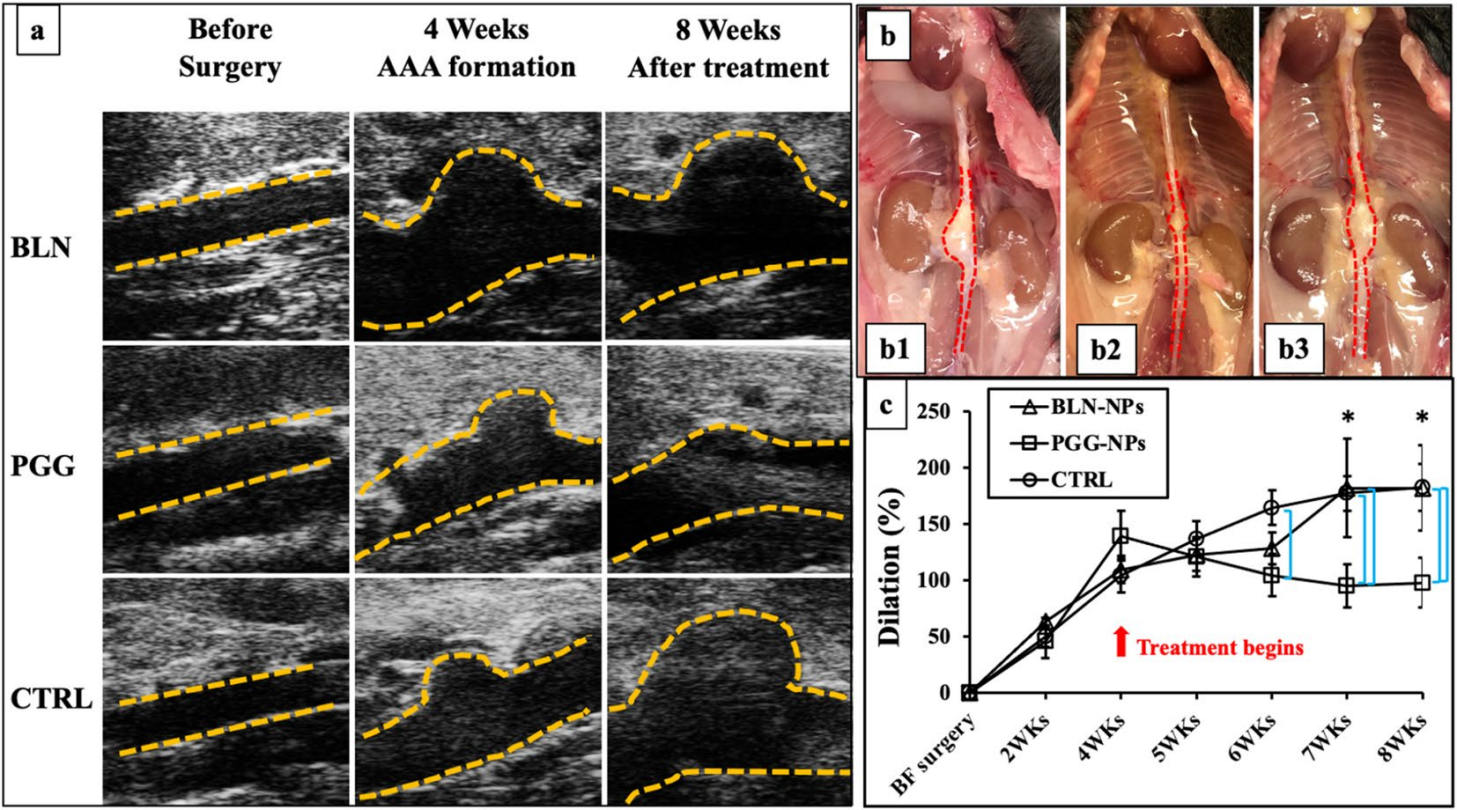
In vivo ultrasound and dilation change. (**a**) Representative B-mode in vivo ultrasound images of abdominal aortas before pump implantation, at 4 weeks when the infusion of Ang II was finished and treatment started, and 8 weeks when the animals were euthanized; (**b**) Gross view of representative aortas from BLN-NPs (b1), PGG-NPs (b2) and control groups (b3) at 8 week; (**c**) Dilation percentage of BLN-NPs group, PGG-NPs group and control group. After therapy PGG-NPs group showed progressive reversal of aneurysmal dilation while control and BLN-NPs groups showed continuous increase in aneurysms. *Indicate timepoint that have dilation percentage significantly different from week 4 (p < 0.1); bars indicate significant difference between groups (p < 0.05).

All Ang-II treated mice showed similar dilation percentages following Ang II infusion. According to the average inner diameters of aneurysms at week four measured and calculated from *in-vivo* abdominal ultrasound, all three groups had more than 100% diametric increase during Ang II infusion. From these, we chose mice that had highest dilation % for use in the treatment group (PGG-NPs). After four more weeks of treatment (2 injections biweekly), the size of the aneurysms became smaller in the PGG-NPs group. The dilation percentages for aneurysms in the BLN-NPs group and the control group kept increasing even without Ang II infusion (Fig. 3a). Gross views of the explanted aortas also showed smaller outer diameters of the suprarenal area in the PGG-NPs group compared to the BLN-NPs group and the control group (Fig. 3b). After treatment, the PGG-NPs group showed a decrease in aortic dilation percentage from 139.16 ± 49.14% to 97.75 ± 49.77%, (p < 0.05) while the aortic diameters kept increasing from 104.10 ± 34.14% to 182.44 ± 46.55% and from 109.38 ± 27.13% to 188.11 ± 84.92% in the BLN-NPs group (p < 0.05) and the control group (p < 0.05), respectively (Fig. 3c).

### Influence of PGG treatment on in vivo circumferential strain (CS) and pulse wave velocity (PWV)

**Figure 4:**
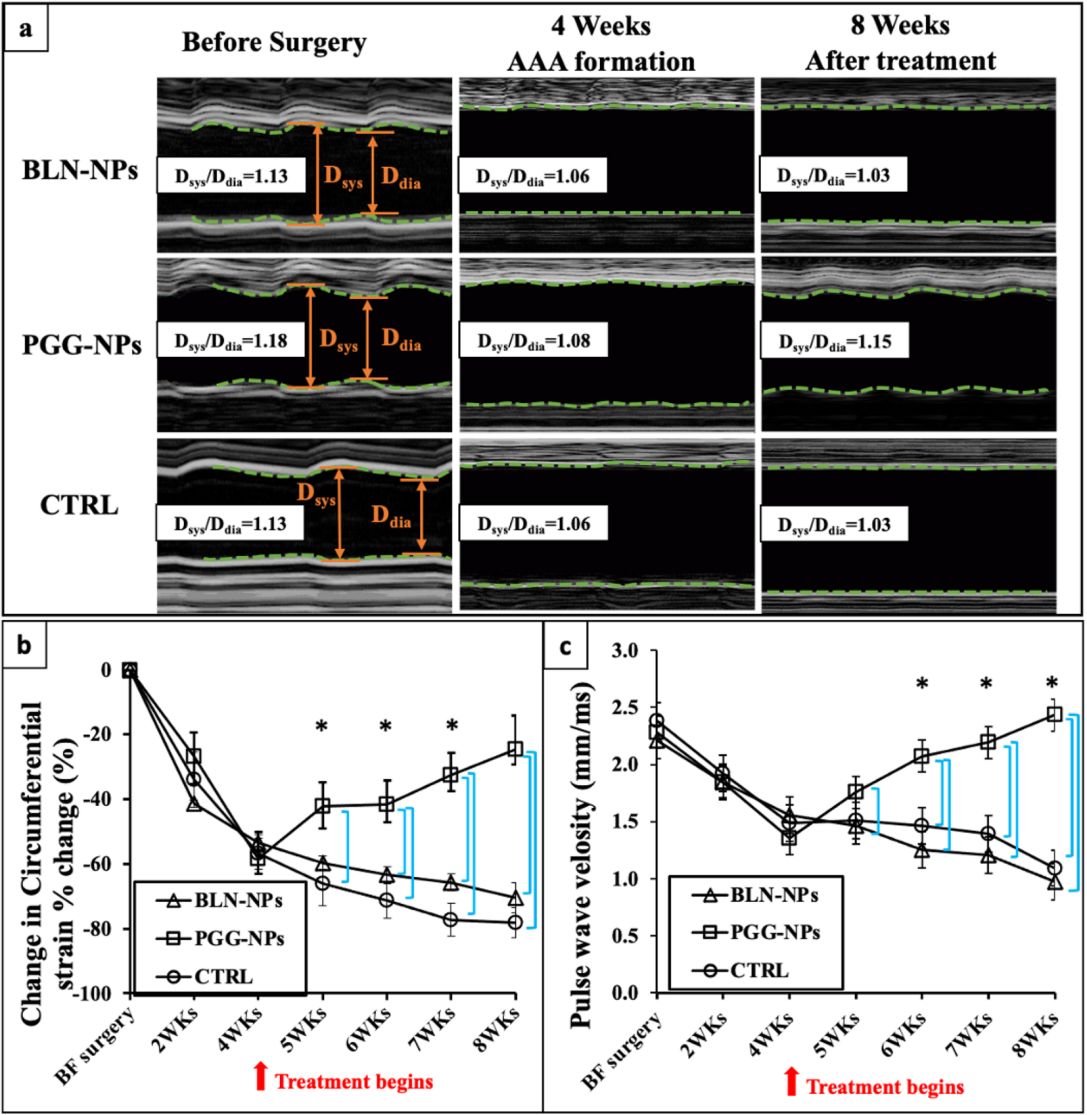
Circumferential strains (CS) and pulse wave velocity (PWV). (**a**) Representative M-mode in-vivo ultrasound images of abdominal aortas before pump implantation, at 4 weeks after infusion of Ang II, and at 8 weeks at the end of therapy, showing the mechanical behavior change within a cardiac cycle for PGG-NPs, BLN-NPs and control groups; (**b**) Percent change of corresponding circumferential Green–Lagrange strains throughout the cardiac cycle at weeks 2 and 4, and during the treatment at weeks 5, 6, 7 and 8. All aneurysmal aortas showed a reduction in CS after formation of the aneurysms. After treatments, PGG-NPs group showed a progressive increase of the CS while the CS of the BLN-NPs group and control group kept decreasing; (**c**) Corresponding PWV at weeks 2 and 4, and during the treatment at weeks 5, 6, 7 and 8. All aneurysmal aortas showed a reduction in the PWV after formation of the aneurysms at 4 week. After treatments, PGG-NPs group showed an increase of the pulse wave velocity, while the pulse wave velocity of the BLN-NPs group and the control group kept decreasing due to further aortic dilation. * indicate timepoint that have dilation percentage significantly different from week 4 (p < 0.05); bars indicate significant difference between groups (p < 0.05).

All mice demonstrated a decrease in systolic to diastolic diameter ratio at the end of the Ang II infusion suggesting stiffening behavior. During four weeks of PGG-NPs treatment, the mechanical behavior of the aortic wall throughout the cardiac cycle started to return towards normal, while in the BLN-NPs group and the control group, the diameter ratios kept decreasing (Fig. 4a). Longitudinal CSs were further calculated to evaluate the change in the distensibility of the aneurysmal aortic wall (Fig. 4b). By the end of the Ang II infusion and prior to NP treatments, the CSs of PGG-NPs, BLN-NPs, and control groups had decreased by 58.25 ± 8.25%, 53.30 ± 2.36%, and 56.85 ± 4.73%, respectively. After four weeks of NPs treatment, aortas treated with PGG-NPs started to regain their distensibility, showing only a 24.50 ± 10.34% CS decrease comparing to the healthy aortas (p < 0.05). However, the CSs for both BLN-NPs-treated group and control group decreased by 70.42 ± 4.73% and 78.14 ± 4.77% of their original values(p < 0.05), respectively, at the endpoint of the treatment.

PWVs were also calculated to evaluate the progression of the AAAs (Fig. 4c). Before the surgery, the PGG-NPs group, BLN-NPs group, and control group had PWVs of 2.28 ± 0.27 mm/ms, 2.21 ± 0.17 mm/ms, and 2.38 ± 0.28 mm/ms, respectively. After the Ang II infusion, the PWVs for the PGG-NPs, BLN-NPs, and control groups dropped to 1.35 ± 0.33 mm/ms, 1.56 ± 0.11 mm/ms, and 1.49 ± 0.19 mm/ms, respectively. Following completion of NP treatment, the PWV of the PGG-NPs group increased to 2.43 ± 0.13 mm/ms (p < 0.05), while the PWV was 0.97 ± 0.12 mm/ms for the BLN-NPs group and 1.09 ± 0.32 mm/ms for the control group (p < 0.05), clearly showing that PGG-NP treatment decreases the diameter of the aorta.

### Histological analysis

**Figure 5:**
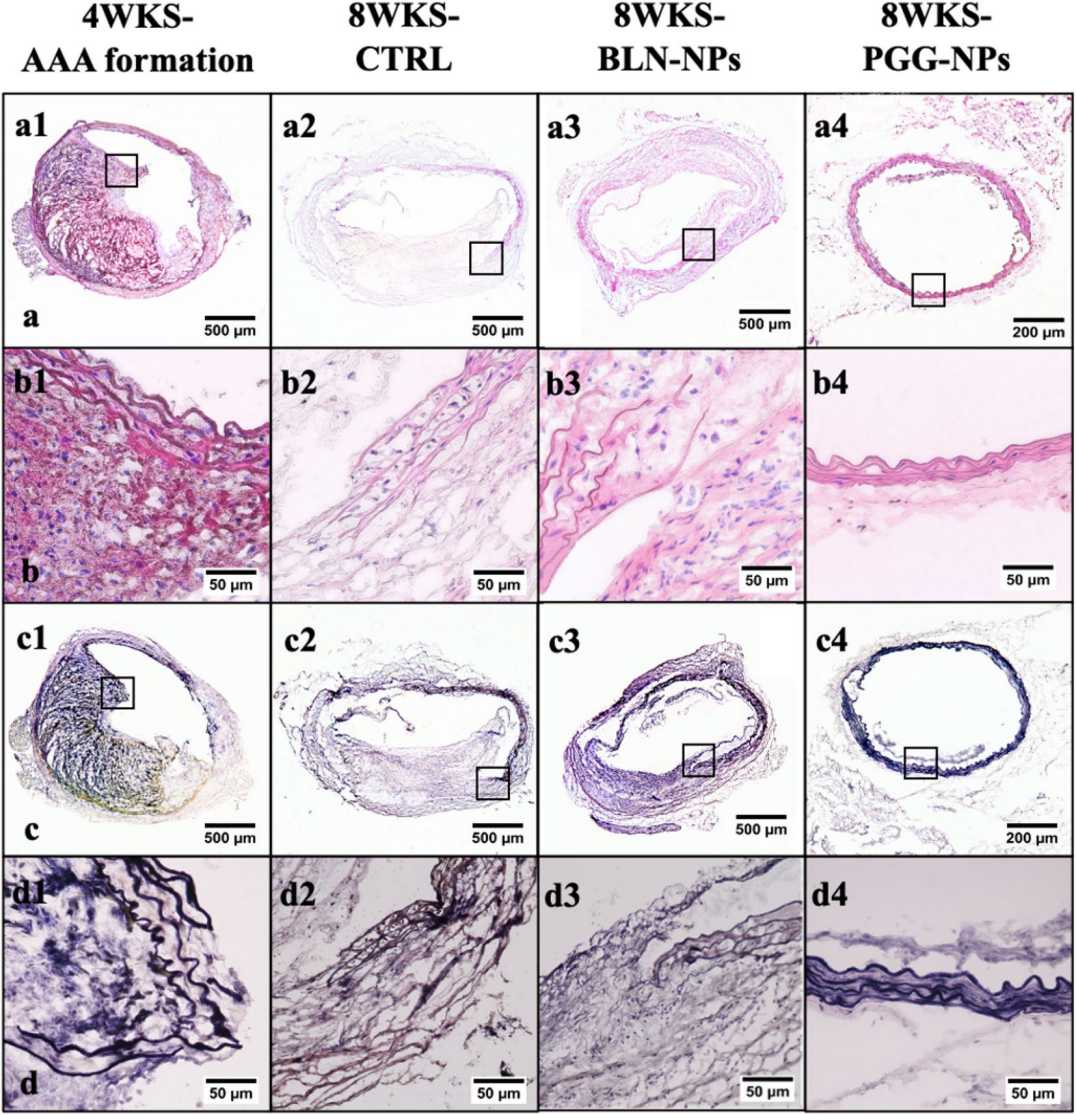
Histological analysis. (**a**,**b**) H&E staining for aneurysms; BLN-NPs, control and PGG-NPs groups are at lower (a1–4) and higher (b1–4) magnifications, showing the morphology and the cell infiltration of the suprarenal aortic tissue. The PGG-NPs group had better morphology and minimal cell infiltration compared to the other groups (the opening shown in a4 and d4 was a vessel branching out from the aorta); (**c**,**d**) VVG staining for aneurysms; BLN-NPs, control and PGG-NPs groups are at lower (c1–4) and higher (d1–4) magnifications, showing the elastin damage within the tissue. The elastic laminae in the PGG-NPs were repaired as compared to before therapy while in other groups they continued to degrade.

H&E and VVG staining of suprarenal aortic sections from all three groups of aneurysms harvested at week four right after the Ang II infusion provided information about the morphology, cellular infiltration, and elastin degradation of the tissues. Stained sections from the 4-week group represented the original status of the aneurysms before treatment. An increase in aortic diameter was observed after aneurysm formation. Massive inflammatory cell infiltrates in the adventitial and medial layers of the aortas and severe elastin degradation in the aortic media were also observed. Comparing the 4-week aneurysms to the sections from BLN-NPs and control groups, the morphology of the tissues from the PGG-NPs group was better than the other three groups, with minimal infiltration of inflammatory cells (Fig. 5a,b). Moreover, VVG staining showed repaired elastin laminae in the PGG-NPs group, while in the other three groups, there was severe degradation of elastin and formation of intraluminal plaques (Fig. 5c,d).

### Assessment of local inflammation

**Figure 6:**
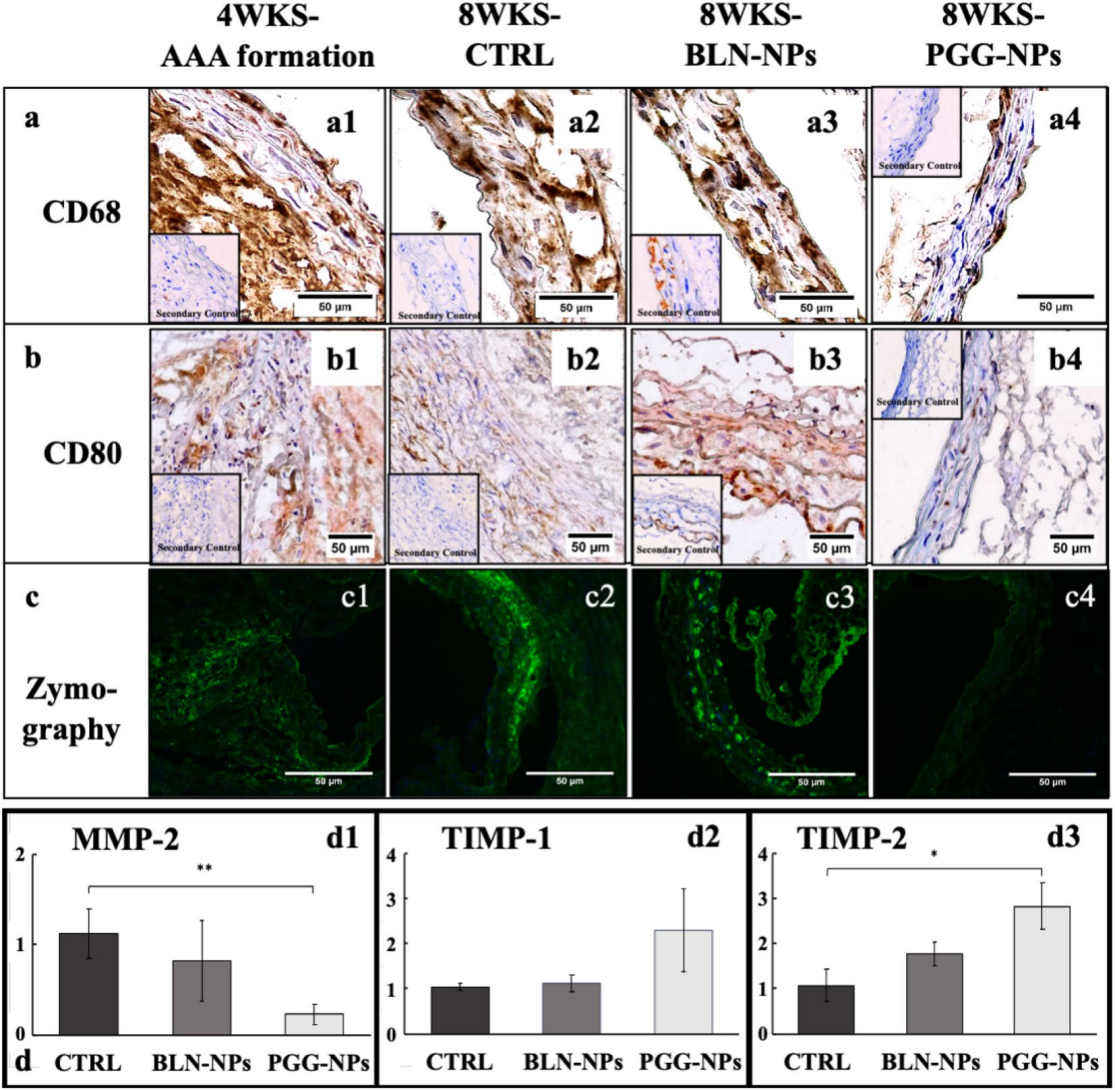
Local inflammatory response analysis of aneurysmal tissues. (**a**,**b**) IHC results for CD68 (a1-a4), staining for pan macrophage infiltration, and CD80 (b1-b4), staining for M1 macrophages, in the suprarenal aneurysmal aortic tissue from 4-week group (before therapy), and at 8-wks for control, BLN-NPs, and PGG-NPs groups. PGG therapy reduced macrophage infiltration in the AAA sites; (**c**) In-situ zymography showing MMP gelatinolytic activity in aneurysmal tissue from 4-week group (before therapy), and at 8-wks control, BLN-NPs and PGG-NPs groups; (**d**) qPCR results of mRNA extracted from aneurysms harvested for MMP-2, TIMP-1 and TIMP-2 expression showing reduction of MMP-2 (*p < 0.05) and increase in TIMP-1 and -2 in PGG group.

IHC staining for CD68 and CD80 in suprarenal aortic sections from PGG-NPs, BLN-NPs, control, and 4-week groups identified macrophages in the aneurysmal tissue. The BLN-NPs, control, and 4-week groups showed intense pan-macrophage (CD68) staining (Fig. 6a1–a3). The PGG-NPs group was also positive for CD68, but the staining was less intense in the adventitia (Fig. 6a4). The BLN-NPs group, control group, and four-weeks group also showed intense M1 macrophage (CD80) staining (Fig. 6b1–b3), while the PGG-NPs group barely showed any staining (Fig. 6b4).

In-situ zymography of suprarenal aortic sections from PGG-NPs, BLN-NPs, and control group visualized matrix metalloproteinase (MMP) activities. The PGG-NPs group showed significant suppression of signal from MMP activities (Fig. 6c4) compared to the BLN-NPs group and control group (Fig. 6c1–3).

qPCR analysis of mRNA from cells in the aneurysmal tissue showed that MMP-2 expression in the PGG-NPs group was significantly lower compared to the control and BLN-NPs groups (p = 0.04) (75–80% reduction) (Fig. 6d1). TIMP-1 and TIMP-2 expression were both upregulated in the PGG-NPs group. The PGG-NPs group showed a 2.2-fold upregulation of TIMP-1 and a higher expression of TIMP-2 (2.8-fold upregulation) compared to the control group (p = 0.07), and a twofold upregulation of TIMP-1 and 1.6-fold upregulation of TIMP-2 compared to the BLN-NPs group (Fig. 6d2–d3). The reduction in MMP expression and increase in TIMP expression in the PGG-NPs group suggest that the balance of matrix homeostasis has shifted from degradation to remodeling.

### Assessment of systemic inflammation

**Figure 7:**
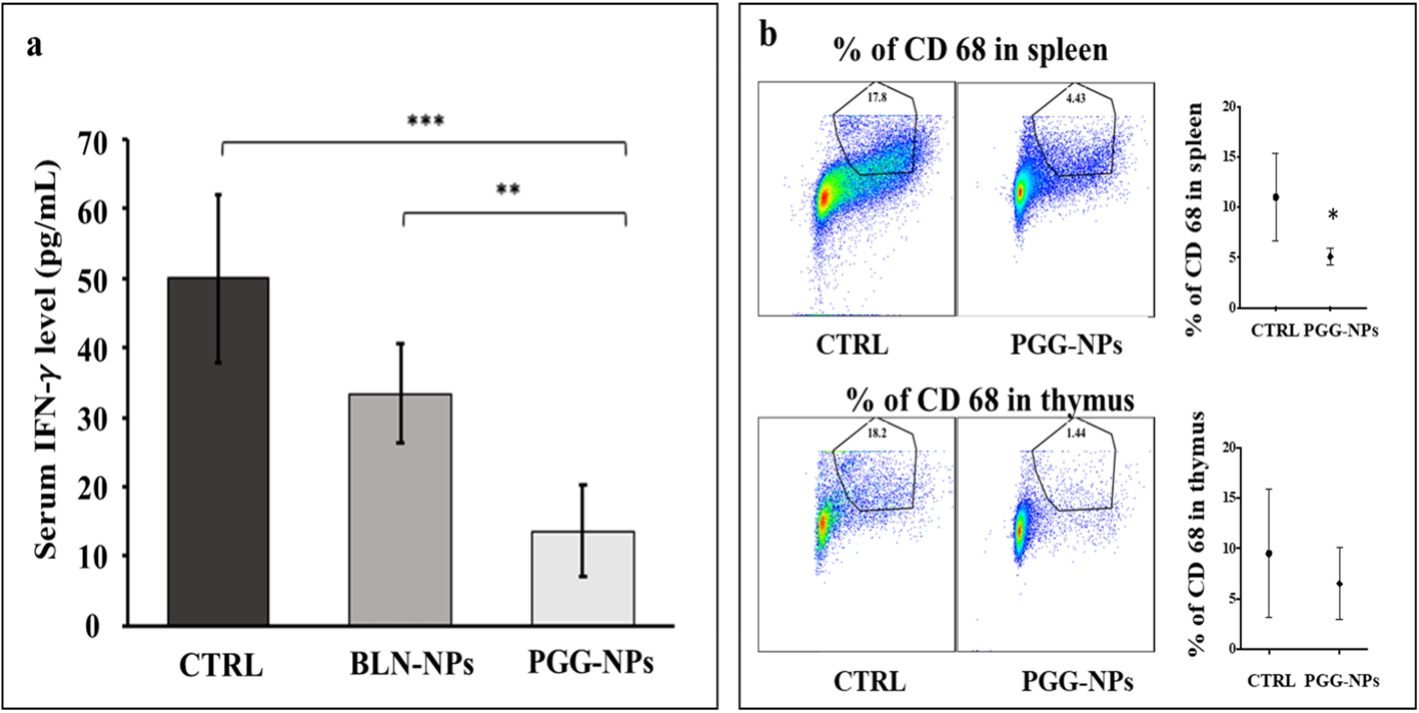
Systemic inflammatory response analysis. (**a**) Serum IFN-γ concentrations of control, BLN-NPs and PGG-NPs groups. PGG-NPs-treated group showed significantly lower serum IFN- γ level than both control and BLN-NPs groups; (**b**) Flow cytometry dot plots and scatter plot for CD 68 positive cells in spleen and thymus from control and PGG-NPs-treated group. The percentage of CD 68 positive cells significantly decreased in spleen in PGG-NPs-treated group in comparison to control group. These results indicate the PGG-NPs therapy decreases systemic inflammatory response. *p < 0.03, **p < 0.05. ***p < 0.01.

Serum IFN-γ levels were measured to evaluate systemic inflammation. The control group had the highest amount of IFN-γ (49.94 ± 14.30 pg/ml) in the serum, followed by the BLN-NPs group (33.34 ± 13.16 pg/ml). The PGG-NPs treated group had the lowest serum IFN-γ concentration (13.55 ± 8.09 pg/ml). The difference in IFN-γ concentration between the BLN-NPs and the control group was not significantly different. However, IFN-γ concentration in the PGG-NPs group was significantly lower than in the control group (p < 0.01) and the BLN-NPs group (p = 0.04) (Fig. 7a). Immunofluorescence staining for CD68 in the spleen showed a significantly decreased percentage of CD68 positive cells (p < 0.03) and a modest decrease in the thymus derived from the PGG-NPs treated mouse group in comparison to the control group (Fig. 7b). This data clearly showed that PGG-NP therapy reduced systemic inflammation.

### Biomechanical assessment of the aorta

**Figure 8:**
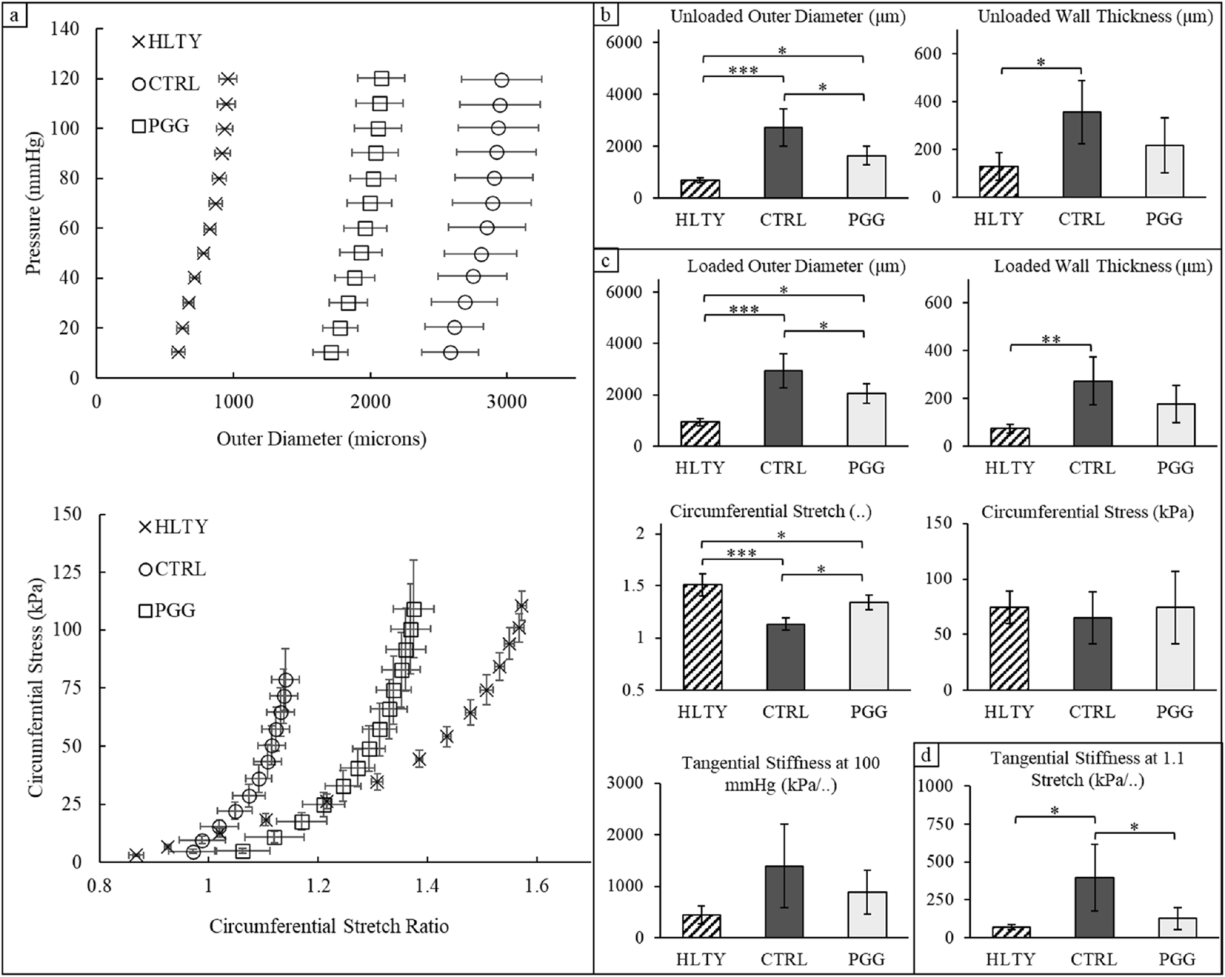
Ex vivo mechanical testing of aneurysmal aortas. Tissues were taken from age-matched healthy (HLTY; n = 5), Ang-II infused aneurysmal controls (CTRL; n = 5), and Ang-II infused aneurysmal pentagalloyl glucose (PGG-NPs; n = 5)-treated animals. (**a**) Pressure-outer diameter and stress-stretch curves measured during continuous inflation- extension testing. Discrete values of tissues in the (**b**) unloaded state, (**c**) physiologically loaded state, and (**d**) at a common stretch ratio. Scatter-plots are mean ± SEM and bar charts (b-d) are mean ± STD. ***p < 0.01, **p < 0.05, *p < 0.1.

Pressure-diameter curves indicated a dramatic increase in the outer diameter for vessels in the aneurysmal control (CTRL) group compared to the healthy (HLTY) group at all inflation pressures (Fig. 8a). In contrast, the pentagalloyl glucose (PGG) treated tissues experienced a decrease in outer diameter compared to the aneurysmal CTRLs but not reaching that of the HLTY group when evaluated at a common pressure of 100 mmHg (loaded; p < 0.001) or 0 mmHg (unloaded; p < 0.001). PGG-treated tissues demonstrated a statistically significant reduction in diameter compared to dilated aneurysmal controls but remained greater than the HLTY group (Fig. 8b,c). Unloaded (p < 0.018) and loaded (p < 0.004) wall thicknesses were only found to be different between HLTY and aneurysmal CTRL vessels with a statistically insignificant trend towards a reduction in thickness in the PGG-NP group. Continuous plots of circumferential stress-stretch suggested a higher stress value for a given stretch in aneurysmal CTRLs. However, no significant differences were found in circumferential stress or tangential stiffness when evaluated at 100 mmHg while the PGG-treated group showed increased levels of stretch compared to the CTRL group (p < 0.001). When evaluated at a low but common stretch ratio of 1.1, controls were found to be stiffer than HLTY while the PGG-treated group revealed a marked improvement through a decrease in tangential stiffness (Fig. 8d; p < 0.050).

## Discussion

Targeted drug delivery to abdominal aortic aneurysms possesses inherent physical challenges due to the complex hemodynamic environment and diverse pathophysiological markers expressed in the tissue. The critical aspects of remodeling occur in the ECM of the aneurysmal tissue, thus it is vital to design a vehicle that can reach and be retained in the ECM to maximize its effect. We chose to work with the Ang II model, one of the most common animal models of AAA, to stimulate spontaneous AAA formation in mice^11^. The formation of AAAs in this model resembles human disease in aspects that include male gender preponderance, the setting of mild hypertension, and the presence of hyperlipidemia^12^. Moreover, the formation of intraluminal thromboses (ILT), which has been commonly seen in this model, is similar to human AAAs.

We and others have shown that elastin degradation presents as a hallmark signal in AAAs^13,14^. Thus, we developed a novel targeting technique with nanoparticles that are conjugated with elastin antibody that only targets the degraded elastin matrix^5,8,15^. We have shown that BSA nanoparticles loaded with DiR dye target these nanocarriers to degrading elastin in-vivo^16^. Concurrent with the targeting data shown by Nosoudi et al. for calcium chloride-induced aneurysm^15^, and Karamched et al. for adenine diet-induced vascular calcification^17^, we demonstrate the targeted delivery of nanoparticles to the aneurysmal site in the Ang II aneurysm model. Importantly, our histological results showed that the DiR-NPs accumulated in the medial layer of the aorta, where the elastin was degrading. It indicated that successful targeting of the NPs was achieved actively under the guidance of the conjugated elastin antibody regardless of the intraluminal plaques. This finding was supported by our previous study suggesting the NPs entered the vessel wall through the vasa vasorum rather than from the aortic lumen^15^.

The change of aneurysmal aortic diameter is one of the most important parameters that could be used to decide the efficacy of AAA pharmacotherapy. Therefore, we measured the inner diameter of the aneurysms at different points throughout the study as part of the evaluation of aneurysm status. The aneurysms all reached at least a 1.5-fold aortic enlargement, a clinical criterion for diagnosing AAAs, by the end of the Ang II infusion. We looked at two critical in vivo assessments of aortic properties, i.e., CS and PWV, to further assess the development of an aneurysm. CS decreased significantly in Ang II infused mice developing aneurysms. It indicated that the aneurysmal aortic walls responded less to blood pressure variation across the cardiac cycle after aneurysm formation. Our observations coincide with other documented results, for example, by Favreau et al.^18^, Trachet et al.^19^, and Phillips et al.^20^ that showed reduced aneurysmal wall distensibility.

In contrast to previous findings of increased PWVs^21–23^, we noticed a significant decrease after 28 days of Ang-II infusion. This follows our hypothesis that increased local aortic diameter at the aneurysmal site increases the time taken by the pulse to travel through the aneurysm. Moreover, the decrease of PWVs resulting from larger aortic diameters outweighed the increase of PWVs caused by the increase of aortic wall stiffness. Nandlall et al. also reported a decrease in PWV after AAA formation in an ApoE/TIMP-1−/− murine model^24^.

After four weeks of treatment, we observed a significant decrease in the size of the PGG-NPs-treated aneurysms. However, aneurysm sizes in the control groups (BLN-NPs and untreated) continued to increase. The PGG-NPs-treated aneurysms also partially restored a healthy level of CS and showed a significant increase in PWVs while the CSs and PWVs continued decreasing in the control groups. In agreement with the findings in a CaCl_2_-induced rat model^15^, we believe that the targeted delivery of PGG restored the mechanical properties of aneurysmal tissue and help regress the established aneurysms. This has been further confirmed through the implementation of ex vivo mechanical testing (Fig. 8) where we found three crucial aneurysm hallmarks were improved following PGG-treatments. These include a reduction in diameter, an increase in mid-wall circumferential stretch, and a decrease in tangential stiffness after PGG treatment. Although tangential stiffness is a linearized measure with limited translatability, when evaluated it at a common but low stretch ratio and found a clear trend towards decreasing stiffness following PGG treatments. This is interesting given that the low stretch region of the stress-stretch curve is dominated by elastin while collagen fibers remain undulated and engage at higher stretch ratios^25–27^. This low-stretch behavior can only be deciphered using ex vivo techniques and supports the theory that PGG-treated tissues undergo significant elastin repair. Likewise, the increased circumferential stretch in PGG-treated tissues enables physiological cardiovascular enhancements through improved Windkessel function and an overall reduction in work across the cardiac cycle. Moreover, our ex vivo data was largely in agreement with the in vivo data showing an improved change in circumferential strain even though both were measured at different reference configurations. By comparison, Patnaik et al.^28^ found a decrease in stiffness moduli with PGG treatments in a porcine model while Pavey et al.^29^ found stiffness to increase in an elastase-induced AAA mouse model. Although PGG has an obvious effect on vascular remodeling, the resultant mechanical properties appear to be treatment and disease model-specific^28^. Despite the inherent complexity of Ang II-induced aneurysms, we used several convenient assumptions to study their mechanical and geometric properties, namely incompressibility, homogeneity, and axial symmetry. We acknowledge that additional metrics would be needed to comprehensively study aneurysmal mechanics, but these were omitted for the sake of simplicity and congruency with the biophysical, histological, and molecular aspects of this work^30^. Nevertheless, the defining feature of aortic aneurysms is the focal increase in diameter, and our PGG-treated tissues demonstrated a significant reduction in diameter compared to the aneurysmal CTRL group. Interestingly, these changes do not manifest as changes in circumferential stress. While aneurysmal tissues exhibited an increase in diameter, they also experienced a concomitant increase in wall thickness. This led to a normalization of circumferential stress that could be attributed to inflammation or to chronic growth and remodeling towards tissue homeostasis^31^. Our PGG-treated animals revealed a small, non-significant reduction in wall thickness compared to the aneurysmal CTRL group and were shown to have a reduced inflammatory response. Collectively, these markers suggest an improved biomechanical phenotype in PGG-treated tissues compared to aneurysmal controls.

We further examined the mechanism of how PGG functioned both systemically and locally to halt and reverse AAA progression. Inflammation in the aortic wall is one of the primary triggers of AAA onset. Chronic inflammatory cell infiltration in the adventitial and medial aortic layers and the upregulation of pro-inflammatory cytokines would result in VSMCs apoptosis and ECM degradation, primarily elastic lamina degradation by proteolytic enzymes such as MMPs and cathepsins^32–34^. Progression of the local aortic inflammatory response requires the enhanced recruitment of inflammatory cells from the circulation^35^. Mellak et al. reported that monocytes from the spleen could be mobilized by the presence of Ang II and contribute to the development of AAA and the occurrence of rupture^36^. According to our flow cytometry results, a significant decrease in CD68-positive cells in the spleen was observed in PGG-NPs-treated group compared with the control group. This observation indicates that PGG could reduce the available monocyte subsets in the system, thus, decreasing aortic macrophage accumulation and attenuating the inflammatory response in the local aneurysmal tissue.

The development of AAAs usually results in an altered systemic level of cytokines such as IFN-γ^37^. The expression of IFN-γ has been reported to be consistently upregulated in the circulation and tissues of patients with AAA^38^, and has been shown to induce production of MMP-9 in macrophages and MMP-2 in SMCs to promote AAA progression^37^. We observed a significantly higher level of IFN-γ in the serum of the BLN-NPs-treated group and the control group compared to the PGG-NPs-treated group. This suggests that PGG could suppress the production of systemic inflammatory markers. It is also noteworthy, that any nanoparticle drug therapy leads to accumulation of NPs in reticuloendothelial (RES) system for clearance. We have previously shown that part of the nanoparticles do end up in liver, spleen, and kidneys^15^. Thus, it is also possible that PGG release there caused systemic effects as well.

At the aneurysmal site, PGG can be thought to have two functions: one is to stabilize the ECM by inhibiting local MMPs, and the second, to increase elastin fiber deposition. It is known that MMP-2 is one of the key players in AAA pathophysiology. MMP-2 expression levels have been shown to increase in AAA conditions in humans^39^. Activated macrophages are responsible for producing MMP-2, which shows increased enzymatic activity when not counterbalanced by the activity of its inhibitors (TIMPs)^3^. TIMPs play a crucial role in reducing aortic aneurysm formation. Results from Eskandari et al.^40^ and Ikonomidis et al.^41^ stressed the importance of TIMP-1 in protecting ECM against aneurysm formation. TIMP-2 inhibits some MMPs, although it can also act as an MMP-2 activator. Thus, it can both promote and attenuate aneurysm formation. Xiong et al.^42^ have reported that the absence of TIMP-2 leads to protection against aneurysm formation, probably by hampering MMP-2 production. However, Aoki et al. showed the protective effect of TIMP-2 in reducing MMP-2 activity^43^. The imbalance of MMP and TIMP activity is primarily responsible for the degradation of collagen and elastin^44^, which are an integral part of the ECM.

Parasaram et al.^45^ have shown that PGG could inhibit MMP activity in vitro and even under acellular conditions (PGG added to development buffer during zymography rather than in cell culture medium). They have also shown that TIMP-1 levels were increased in rat pulmonary fibroblasts treated with PGG in-vitro. In our study, we have shown that PGG-NP treatment inhibited MMP activity using in situ zymography of an aneurysmal site. PGG-NPs-treated aortas showed diminished MMP activity compared to control and BLN-NP group counterparts. Moreover, we observed that PGG-NPs treatment significantly reduced MMP-2 expression while upregulating TIMP expression in the aneurysmal tissue compared with the control group. PGG treatment has been suggested to help achieve a beneficial protease/anti-protease balance in the aneurysmal tissue.

We have shown earlier that PGG binds strongly to elastin and protects it from degradation by enzymes^13^. Furthermore, we have shown^45,46^ that PGG also helps to increase elastin deposition in cell cultures, possibly by binding and coacervating tropoelastin molecules. This process helps lysyl oxidase (LOX) to crosslink tropoelastin at the site of the aneurysm. While the amount of tropoelastin produced by cells remains similar in control and PGG-treated cells, PGG-treated cells have more elastic fibers laid down in the ECM. Our VVG staining results indicate the replenishment of elastin fibers that are lost during aneurysm development in the PGG-NP treated group. Restoration of elastin fibers at the aneurysmal site could explain the changes observed in the biomechanical properties of aortas in this group.

By stabilizing and regenerating elastin fibers in the ECM, PGG-NP treatment further ameliorated inflammation in the AAAs. Some products of ECM degradation at the site of injury, such as elastin fragments (EFs) or elastin degradation products (EDPs), are chemotactic to macrophages^47^. Thus, suppression of elastin degradation may have further led to suppression of macrophage activity in the area and significant suppression of MMP activity in the PGG-NP group. We have shown using IHC that the levels of CD68-stained macrophages decreased significantly with PGG treatment. Furthermore, CD80 staining revealed a complete absence of pro-inflammatory (M1) macrophages at the aneurysmal site. We thus conclude that PGG works by both inhibiting MMPs and by restoring elastin in the aorta, which stops the recruitment of macrophages, thereby restoring a “normal” microenvironment at the aneurysmal site. It might exert systemic effect as well as shown above that can further aid suppression of inflammatory cell recruitment.

## Conclusion

In the present study, we investigated the efficacy of PGG-NPs to treat AAA in the Ang II infusion-induced mouse model. PGG-NPs treated mice demonstrated reversal of many of the pathological features of AAAs such as a decrease in aortic diameter, improved tissue morphology, restoration of elastin fibers in the ECM, decreased local and systemic inflammation, and the improvement in overall mechanical properties. Thus, targeted delivery of PGG-NPs serve as a promising therapeutic strategy for reversing already established AAAs.

## Supplementary Information

Supplementary Figure 1.
