# Author Correction: Systemic delivery of targeted nanotherapeutic reverses angiotensin II‑induced abdominal aortic aneurysms in mice

**DOI:** 10.1038/s41598-021-95395-8

**Published:** 2021-07-30

**Authors:** Xiaoying Wang, Vaideesh Parasaram, Saphala Dhital, Nasim Nosoudi, Shahd Hasanain, Brooks A. Lane, Susan M. Lessner, John F. Eberth, Naren R. Vyavahare

**Affiliations:** 1 Department of Bioengineering, Clemson University, 501 Rhodes Engineering Research Center, Clemson, SC 29634 USA; 2 Biomedical Engineering, College of Engineering & Computer Sciences, Marshall University, Huntington, WV USA; 3 Department of Cell Biology and Anatomy, University of South Carolina School of Medicine, Columbia, USA

Correction to: *Scientific Reports* 10.1038/s41598-021-88017-w, published online 21 April 2021

The original version of this Article omitted an affiliation for Nasim Nosoudi. The correct affiliations are listed below.

Department of Bioengineering, Clemson University, 501 Rhodes Engineering Research Center, Clemson, SC 29634, USA.

Biomedical Engineering, College of Engineering & Computer Sciences, Marshall University, West Virginia, USA.

The original Article has been corrected.

