# Supplementary figures and images for: Systemic delivery of targeted nanotherapeutic reverses angiotensin II-induced abdominal aortic aneurysms in mice
Source: Sci Rep. 2021 Apr 21;11:8584. doi: 10.1038/s41598-021-88017-w (PMC8060294; doi:10.1038/s41598-021-88017-w)

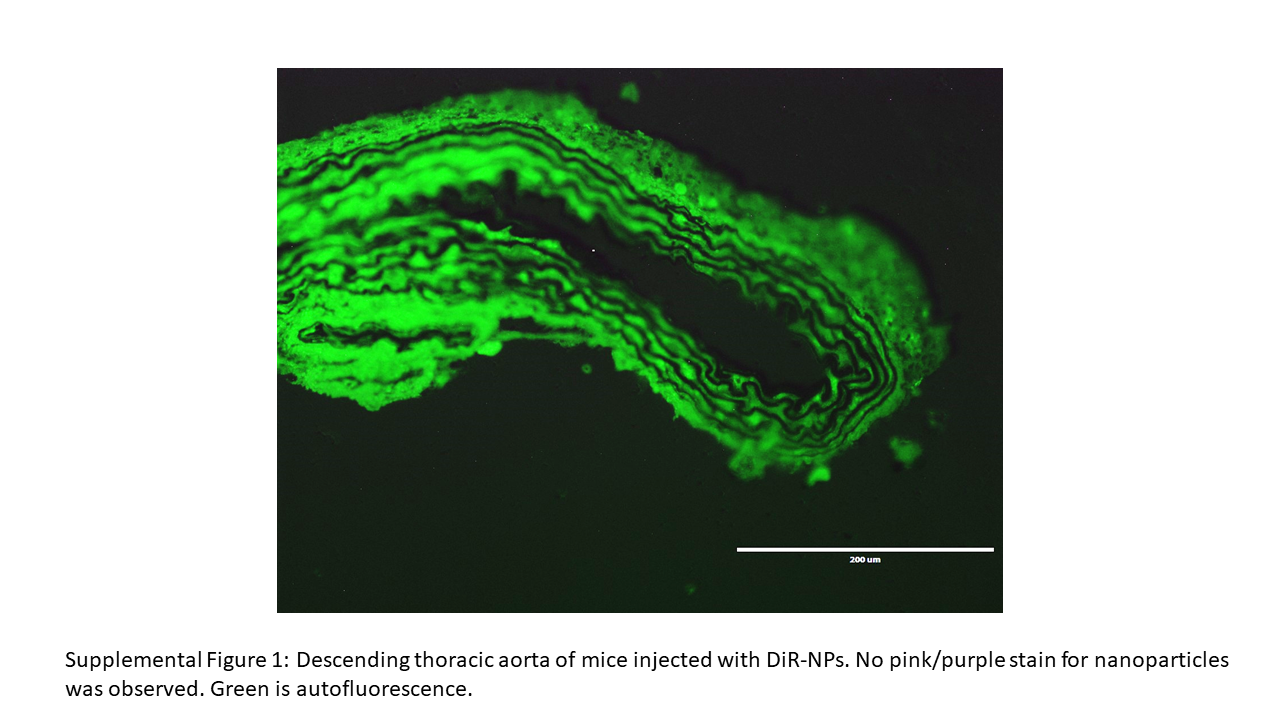

Supplement: Supplementary file 1 — Supplementary Figure 1. [file 41598_2021_88017_MOESM1_ESM.tif]
